# Patient Complications and Device Issues Associated With FDA-Approved Intragastric Balloons Available in the USA: A Maude Database Study

**DOI:** 10.1007/s11695-024-07128-1

**Published:** 2024-03-11

**Authors:** Morgan C. Goodman, Patrick Chang, William Minteer, Denis Nguyen, Kalpana Gopalkrishnan, Jennifer Phan

**Affiliations:** 1 Division of Gastrointestinal and Liver Diseases, Keck School of Medicine of the University of Southern California, Los Angeles, CA USA; 2 Department of Medicine, Keck School of Medicine of the University of Southern California, Los Angeles, CA USA

**Keywords:** Intragastric balloons, Obesity treatment, Weight loss, Endoscopy

## Introduction

Intragastric balloons (IGB) are an effective non-surgical weight loss option for patients with obesity (body mass index 30–35 kg/m^2^) who are not surgical candidates [1]. There are four FDA-approved IGBs; however, only two—Spatz3 (Spatz FGIA, Great Neck, NY) and Orbera (Apollo Endosurgery, Austin, TX, USA)—are currently available within the USA. This study characterizes device issues and patient complications involving these IGBs, using post-marketing surveillance data from the FDA Manufacturer and User Facility Device Experience (MAUDE) database between December 2020 and October 2023. Our analysis provides an update to the 2021 Ramai et al. publication [2] on IGB adverse events reported to MAUDE and presents new data on Spatz3, not FDA approved until October 2021.

## Methods

The MAUDE database (https://www.accessdata.fda.gov/scripts/cdrh/cfdocs/cfmaude/search.cfm) tracks adverse event reports involving FDA-approved medical devices. Reports contain information on the device, manufacture, and event details, including report date, device problems, patient complications, and free-texted narratives.

MAUDE was queried for entries on Orbera and Spatz3 IGBs submitted between December 2020 and September 2023. Search terms included Spatz3, Spatz FGIA, and Orbera, Apollo Endosurgery.

## Results

During the study period, 728 cases (Orbera = 354, Spatz3 = 374) with 1099 device issues and 1021 patient complications were reported to MAUDE.

**Fig. 1:**
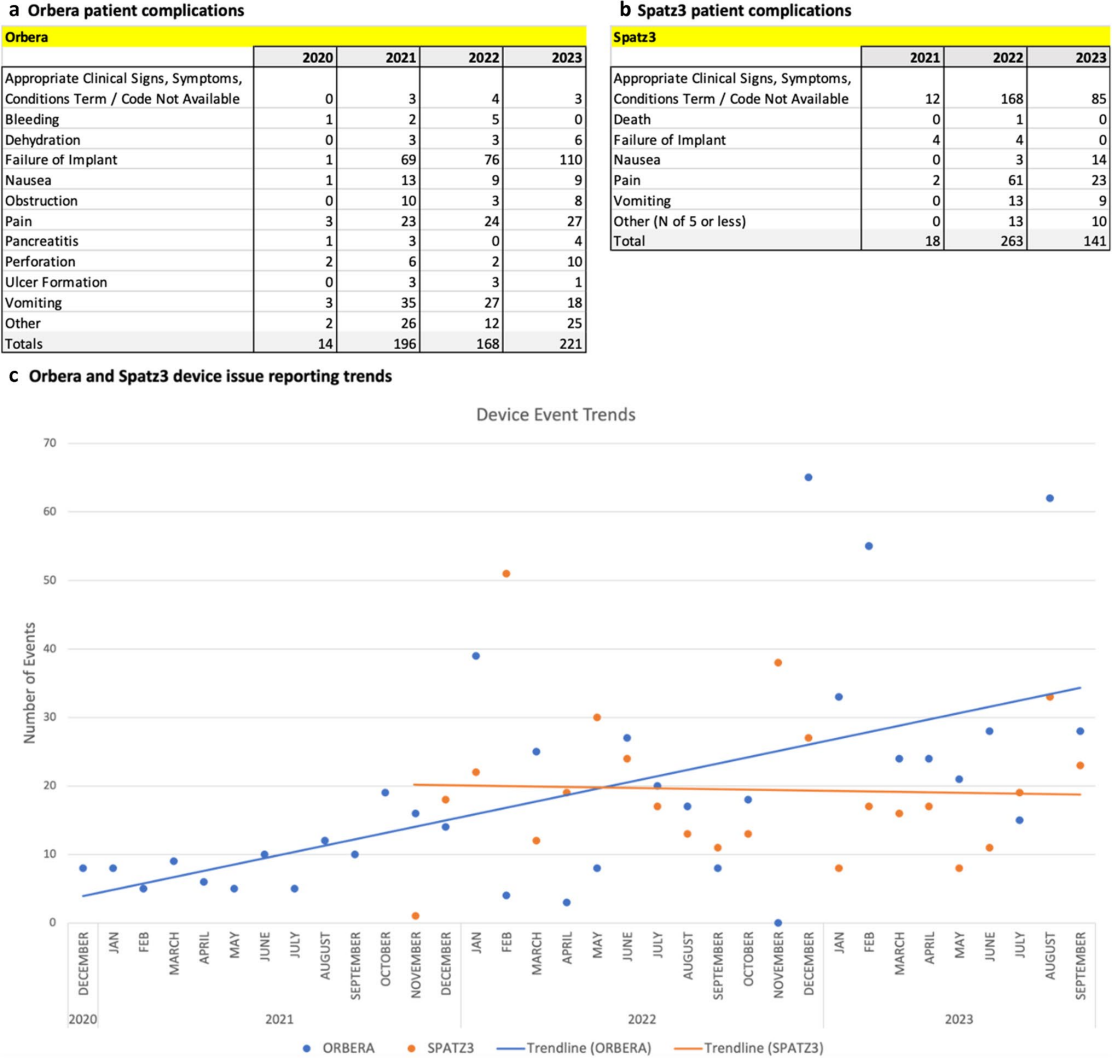
Temporal trends. Patient complications reported by year for Orbera (**a**) and Spatz3 (**b**). Device issue reports by month (**c**) with trendlines (Orbera = blue, Spatz3 = orange

Orbera IGBs, FDA approved in 2005, had 651 device issues between December 2020 to September 2023 with reports increasing each year since 2021 (Fig. 1a–c). Spatz3 IGBs had 448 device issues reported between November 2021 to September 2023. A similar number of device issues were reported during the first year after FDA approval in October 2021 (*N* = 231) and the subsequent 11 months (*N* = 217).

Balloon deflation accounted for 49% and 63% of all Orbera and Spatz3 device issues, respectively. Leak (*N* = 140, 22%) and balloon inflation problems (*N* = 64, 10%) for Orbera IGBs and patient-device incompatibility (*N* = 45, 10%) and device migration or expulsion (*N* = 41, 9%) for Spatz3 IGBs, were the next most reported device issues (Table 1a). Report narratives described balloon hyperinflation (*N* = 29, 64%), gastric ulcer formation (*N* = 6, 13%), and symptomatic intolerance (*N* = 5, 11%) as leading reasons for patient-device incompatibility. Among this group, 10 patients required hospitalization and 22 required device removal or replacement. Hyperinflation accounted for 50% of 24 patient-device incompatibility issues, with 6 noting device removal or replacement was required.

**Table 1:**
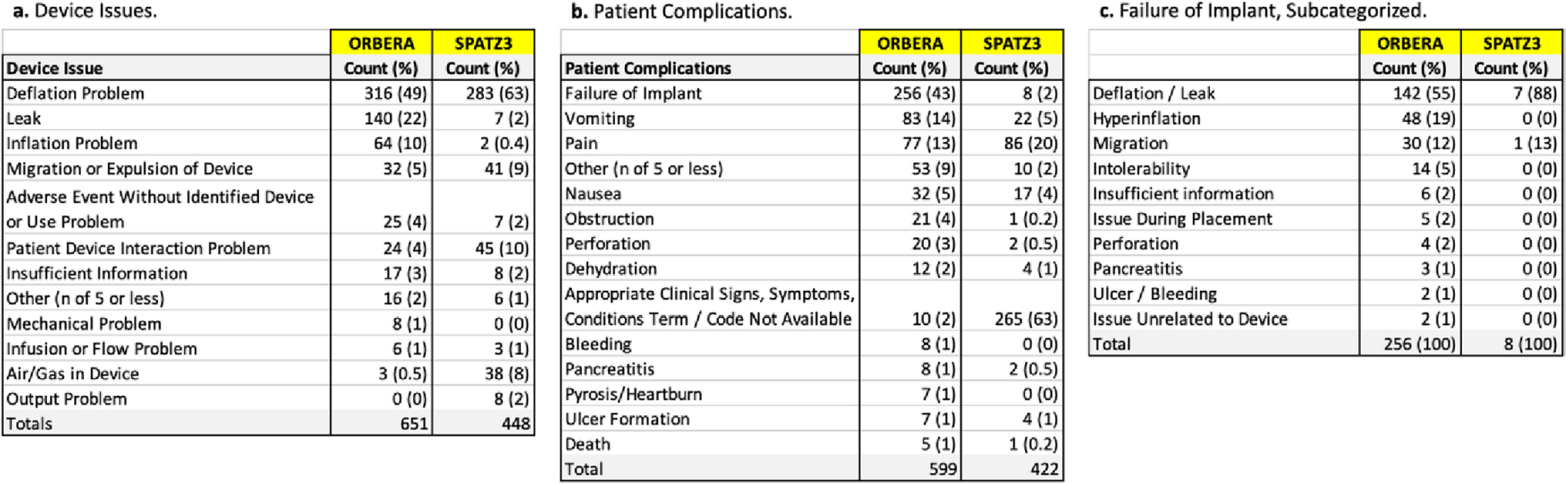
Total device issues (a) and patient complications (b) for Orbera and Spatz3 IGBs reported over the study period; categories with fewer than 5 counts were combined into “other.” (c) “Failure of implant” patient complications, subcategorized based upon narrative descriptions

A total of 599 patient complications involving Orbera and 422 involving Spatz3 IGBs were reported (Table 1b). Failure of implant (*N* = 256, 43%), vomiting (*N* = 83, 14%), pain (*N* = 77, 13%), nausea (*N* = 32, 5%), obstruction (*N* = 21, 4%), and perforation (*N* = 20, 3%) were leading Orbera-related patient complications. Report narratives detail IGB leak or deflation (*N* = 142, 55%), hyperinflation (*N* = 48, 19%), and migration or expulsion (*N* = 30, 12%) as primary causes of Orbera implant failure (Table 1c). For Spatz3, appropriate clinical signs, symptoms, conditions term or code not available (*N* = 265, 63%) was the most frequently indicated patient complication, followed by pain (*N* = 86, 20%), vomiting (*N* = 22, 5%), and nausea (*N* = 17, 4%). IGB leak or deflation (*N* = 215, 81%) is the leading device issue underlying “Appropriate Clinical Signs/Code Not Available” according to narrative reports. Ulcer formation and pancreatitis each accounted for ≤ 1% of all patient complications for either IGB. Six deaths were reported during the study period (Orbera = 5, Spatz3 = 1). Five were related to perforation (Orbera = 4, Spatz3 = 1), with few additional details provided, and one did not disclose a cause.

## Conclusion

This study provides an accounting of FDA approved, market-available IGB device issues and patient-related complications. Randomized controlled trials of Orbera [3] and Spatz3 [4] found high rates of nausea (Orbera = 86.9%, Spatz3 = 90.4%), vomiting (Orbera = 75.6%, Spatz3 = 71.7%), and abdominal pain (Orbera = 75.6%, Spatz3 = 60.4%) after IGB placement. Collectively, these were also the most frequently reported symptoms to MAUDE for either IGB; however, each accounted for a smaller than expected proportion of overall reports when considering prior study findings. These differences may highlight reporting bias resulting from event underreporting due to voluntary reporting requirements.

While clinical trials involving Orbera and Spatz3 found low rates of spontaneous deflation between 0 and 1.66% [3, 5], over half of all reported device issues in this study was related to balloon deflation or leaking. A majority of deflations/leaks were detected by the patient due to change in urine color or during device retrieval; few were identified because of suboptimal weight loss. Extending IGB use beyond recommendations or non-adherence with proton pump inhibitors can increase the risk of deflation [6] and may contribute to deflation reports. Balloon hyperinflation, another rarely cited IGB complication [7], was frequently described within narrative reports for patient-device incompatibility issues for both IGBs (Orbera = 64%, Spatz3 = 50%). While most reports lack manufacturer verification or root cause analysis, these findings suggest that spontaneous deflation and hyperinflation may be more common than previously described.

Study limitations relate to the quality and completeness of MAUDE reports. Nevertheless, MAUDE data provides insights on complications encountered in clinical practice, playing an important role in monitoring devices, such as the recently approved Spatz3. Findings from this study may inform patient-provider discussions of IGB complications and have identified spontaneous deflation and hyperinflation as potential areas for further study.

## Data Availability

The data that support the findings of this study are openly available at MAUDE - Manufacturer and User Facility Device Experience: https://www.accessdata.fda.gov/scripts/cdrh/cfdocs/cfmaude/search.cfm.
